# Exploring skeletal muscle tolerance and whole‐body metabolic effects of FDA‐approved drugs in a volumetric muscle loss model

**DOI:** 10.14814/phy2.15756

**Published:** 2023-06-18

**Authors:** Shefali R. Bijwadia, Christiana J. Raymond‐Pope, Alec M. Basten, Mason T. Lentz, Thomas J. Lillquist, Jarrod A. Call, Sarah M. Greising

**Affiliations:** ^1^ School of Kinesiology University of Minnesota Minneapolis Minnesota USA; ^2^ Department of Physiology and Pharmacology University of Georgia Athens Georgia USA; ^3^ Regenerative Bioscience Center University of Georgia Athens Georgia USA

**Keywords:** formoterol, muscle function, neuromusculoskeletal injury, nintedanib, skeletal muscle injury

## Abstract

Volumetric muscle loss (VML) is associated with persistent functional impairment due to a lack of de novo muscle regeneration. As mechanisms driving the lack of regeneration continue to be established, adjunctive pharmaceuticals to address the pathophysiology of the remaining muscle may offer partial remediation. Studies were designed to evaluate the tolerance and efficacy of two FDA‐approved pharmaceutical modalities to address the pathophysiology of the remaining muscle tissue after VML injury: (1) nintedanib (an anti‐fibrotic) and (2) combined formoterol and leucine (myogenic promoters). Tolerance was first established by testing low‐ and high‐dosage effects on uninjured skeletal muscle mass and myofiber cross‐sectional area in adult male C57BL/6J mice. Next, tolerated doses of the two pharmaceutical modalities were tested in VML‐injured adult male C57BL/6J mice after an 8‐week treatment period for their ability to modulate muscle strength and whole‐body metabolism. The most salient findings indicate that formoterol plus leucine mitigated the loss in muscle mass, myofiber number, whole‐body lipid oxidation, and muscle strength, and resulted in a higher whole‐body metabolic rate (*p* ≤ 0.016); nintedanib did not exacerbate or correct aspects of the muscle pathophysiology after VML. This supports ongoing optimization efforts, including scale‐up evaluations of formoterol treatment in large animal models of VML.

## 1 INTRODUCTION

Volumetric muscle loss (VML) is an orthopedic injury involving the loss of skeletal muscle mass and function, commonly associated with battlefield blast trauma and surgical ablations (Cross et al., 2011; Owens et al., 2007, 2008). Despite persistent functional deficits, which are a significant medical and quality of life issue, there are currently no clinical standards for managing the soft tissue loss after VML (Grogan et al., 2011; Tanaka et al., 2017). The lack of muscle regeneration, in part, drives chronic functional loss, and the muscle remaining after VML is known to lack the same plasticity and adaptability as uninjured muscle (Aguilar et al., 2018; Garg et al., 2014; Greising et al., 2018; McFaline‐Figueroa et al., 2022). Mechanistically this could be due to several factors, including prolonged inflammation, mitochondrial structural damage, denervation, lack of physical activity/loading, loss of myogenic signaling, and fibrosis (Corona et al., 2013; Hoffman et al., 2022; Larouche et al., 2022; Sorensen et al., 2021; Southern et al., 2019). Another important and recently identified aspect of the sequelae of VML injury is the alteration in whole‐body and local muscle metabolism, (Dalske et al., 2021; McFaline‐Figueroa et al., 2022; Raymond‐Pope et al., 2023) which may underlie the inability of the muscle remaining to recover function. Therefore, ongoing evaluations into interventions that could stimulate regeneration by targeting mechanistic limitations could be transformative to promote functional improvements in VML‐injured muscle.

Potential therapies, including regenerative and/or rehabilitative approaches, have shown a modest ability to result in recovery of muscle function (i.e., functional recovery) in preclinical models of VML (see for review: Grasman et al., 2015; Greising et al., 2019). Recent efforts have used a drug‐repurposing approach to improve the quality and the function of the remaining muscle and promote metabolic changes. Several FDA‐approved pharmaceutical therapies have been tested in preclinical models of VML to inhibit inflammation and fibrotic tissue accumulation, including losartan, nintedanib, and tacrolimus (Greising et al., 2019). Nintedanib, a tyrosine kinase inhibitor that binds vascular endothelial growth factor (VEGFR), platelet‐derived growth factor (PDGFR), and fibroblast growth factor receptors (FGFR), is FDA‐approved for reducing the progression of idiopathic pulmonary fibrosis (Rivera‐Ortega et al., 2018; Wollin et al., 2015). It is clear that the pathologic deposition of fibrotic tissue after skeletal muscle injuries, such as VML, may limit regrowth and distribution of myofibers (Delaney et al., 2017; Gardner et al., 2020; Wynn, 2008). Nintedanib's anti‐fibrotic effects at the muscle are able to mitigate fibrosis in *mdx* mice (Pinol‐Jurado et al., 2018) and decrease muscle stiffness and collagen content in a pig model of VML (Corona et al., 2020). However, further dosing optimization and evaluation of regenerative and functional outcomes are warranted. It is possible that by chronically mitigating fibrosis through treatment, myogenic signaling and recovery of muscle function following traumatic muscle injury will be maintained long‐term.

While the fibrotic and inflammatory aspects following VML injury have been a primary focus of investigation, understanding, and targeting the metabolic impairments after VML has only been more recently investigated. One example of a treatment that promotes mitochondrial biogenesis through *PGC‐1α* activation and stimulates anabolic pathways for protein synthesis (e.g., PI3k/Akt/mTOR) is β_2_ adrenergic receptor agonists. Recently, the β_2_ adrenergic receptor agonist, formoterol, has shown promise for improving whole‐body and local muscle metabolism following VML injury (McFaline‐Figueroa et al., 2022; Raymond‐Pope et al., 2023). Formoterol, a long‐acting β_2_ adrenergic receptor agonist and an FDA‐approved inhalant therapy used as a bronchodilator for obstructive lung diseases, improves mitochondrial function and diurnal metabolic flexibility, the ability to transition between substrates used as fuel, chronically following VML (Faulds et al., 1991; Raymond‐Pope et al., 2023). Formoterol also improves muscle mass in conditions of muscular dystrophy and spinal cord injury, while also noted to reduce inflammation following cryolesion muscle injury (Conte et al., 2012; Harcourt et al., 2007; McFaline‐Figueroa et al., 2022; Raymond‐Pope et al., 2023; Ryall et al., 2006). Similarly, formoterol shows promise for improving muscle mass and function chronically following VML, demonstrating higher maximal isometric torque and mitochondrial oxygen consumption rate compared to VML injuries left to the natural sequela of injury (McFaline‐Figueroa et al., 2022; Raymond‐Pope et al., 2023). It is possible the improved muscle mass and function following chronic formoterol administration could be enhanced with the addition of leucine, an essential branched‐chain amino acid. Leucine is known to facilitate muscle protein synthesis by stimulating mTOR, (Anthony et al., 2000; Dyachok et al., 2016) while inhibiting proteolysis and autophagy (Son et al., 2020). When combined, formoterol and leucine may synergistically activate the mTOR pathway to promote functional muscle recovery chronically following VML.

This research aims to further explore promising pharmacologic options targeting fibrosis, inflammation, and metabolic dysfunction after VML to establish potential clinical options to maximize return of function for patients with VML. To evaluate this, the effects of two relevant pharmaceuticals, an anti‐fibrotic, nintedanib, and a muscle hypertrophy stimulant, formoterol combined with leucine, were evaluated following VML. The first cohort of mice was used to optimize pharmacologic responses in the absence of concomitant injury. Second, a cohort of VML‐injured mice were used to evaluate the effects of the treatments after injury.

## 2 MATERIALS AND METHODS

### 2.1 Ethical approval and study design

All experimental protocols and animal care guidelines were approved by the Institutional Animal Care and Use Committee and the University of Minnesota (#2008‐38365A); in compliance with the Animal Welfare Act, the Implementing Animal Welfare Regulations, and in accordance with the principles of the Guide for the Care and Use of Laboratory Animals. Mice were housed on a 12‐h light–dark cycle (light phase begins at 06:00) with ad libitum access to chow and water.

Adult (12‐week‐old; total *n* = 72) male C57BL/6J mice (Jackson Laboratories #000664; Bar Harbor, ME) were used in all studies. While this study only included female mice, future work should evaluate any sex‐specific differences to overcome this limitation. Prior to any study procedures being performed, mice were given a one‐week acclimation period. Mice were randomly allocated to two cohorts. First, to optimize dosing, mice were randomized to one of the two pharmaceutical treatments, each separated into a high‐ (*n* = 8) and low‐dose (*n* = 8) group, or no treatment (i.e., vehicle control; *n* = 8). Following optimization of skeletal muscle mass and myofiber cross‐sectional area, the second cohort of mice underwent unilateral VML surgery to the posterior muscle compartment (gastrocnemius, soleus, plantaris muscles) or served as injury naïve controls. The mice that underwent VML surgery were randomly assigned to a pharmaceutical treatment immediately following VML or no treatment. Mice were excluded from the protocol and analyses in the events of death prior to terminal harvest, weight loss ≥20% of initial total body weight, and/or failure to thrive following gavage treatment. Three mice in each cohort (*n* = 6 total) were removed from the study and no data from these mice were used in the work. At the terminal time point (4 or 8 weeks), while deeply anesthetized via inhaled isoflurane (~2.0%), blood was collected via cardiac puncture and serum was stored at −20°C, and skeletal muscles were harvested, weighed, frozen, and stored at −80°C for later analyses. The extensor digitorum longus (EDL), soleus, and/or middle third of the gastrocnemius (i.e., encompassing the VML defect) muscles were saved for histological analyses, while the tibialis anterior (TA) and remaining proximal and distal portions of the gastrocnemius were saved for biochemical analyses. All mice were euthanized with pentobarbital (>100 mg/kg; s.q.). For all experimental procedures and analyses, investigators were blinded.

### 2.2 Surgical model of VML injury

As previously described, a unilateral VML injury to the posterior muscle compartment was surgically created (Greising et al., 2018; Southern et al., 2019). Mice received preoperative buprenorphine SR (1 mg/kg s.q.) and were anesthetized via inhaled isoflurane (~2.0%) for the duration of the surgery, under aseptic surgical conditions. A posterior‐lateral incision was made to reveal the posterior compartment. Blunt dissection separated the fascia and hamstring muscles from the gastrocnemius muscle. A metal spatula was placed under the posterior muscles, posterior to the tibia at the muscle mid‐belly. Approximately 15% of the muscle was removed using a 4‐mm biopsy punch (18.5 ± 5.0 mg). The skin was closed. Postoperatively, appearance, behavior, weight, waste outputs, appetite, and wound healing were monitored.

### 2.3 Pharmacologic treatments

Mice in treatment groups were administered nintedanib or formoterol plus leucine. During the initial optimization experiments, two doses were evaluated for each treatment. Nintedanib (Cayman Chemical #11022) was delivered at 6 mg/kg/day or 60 mg/kg/day via daily gavage at a volume of 0.01 mL/g body weight (0.9% saline solution and 0.01% dimethyl sulfoxide). Formoterol (Sigma #1286107) plus leucine was delivered via additive to the standard chow of mice at 0.3 mg/kg/day plus 8 g/kg/day or 3 mg/kg/day plus 24 g/kg/day, respectively. Doses were selected based on doses previously evaluated in models of liver and lung fibrosis, and muscular dystrophy, (Pinol‐Jurado et al., 2018; Wollin et al., 2014, 2015, 2020) and in models of cancer cachexia, spinal cord injury, and VML injury (Busquets et al., 2004; McFaline‐Figueroa et al., 2022; Raymond‐Pope et al., 2023; Scholpa et al., 2021; Scholpa, Simmons, et al., 2019). Untreated mice were fed non‐enriched but matched macro‐ and micro‐nutrient content as the standard chow (Mod LabDiet #5053; LabDiet, St. Louis, MO) and received 0.01 mL/g body weight of 0.9% saline, via gavage weekly. For the intervention following VML, mice received either nintedanib at 6 mg/kg/day or formoterol plus leucine at 3 mg/kg/day plus 24 g/kg/day; based on results of the initial optimization experiments. Treatments began immediately following VML injury and were continued for 8 weeks.

### 2.4 Whole‐body metabolism and physical activity

Physical activity and whole‐body metabolism data were collected 6 weeks post‐VML using Columbus Instruments' Comprehensive Lab Animal Monitoring Systems metabolic chambers and data examination tool (Clax v2.2.15) as previously described (Basten et al., 2023; Dalske et al., 2021; Raymond‐Pope et al., 2023). Briefly, mice were housed singly for 48 h, consisting of a 24‐h period of acclimation and of data collection. Metabolic rate (MR), respiratory exchange ratio (RER), and total ambulation distance data were collected. Analysis was performed in MATLAB to quantify average MR and RER over 24–h and 12‐h active and inactive periods.

### 2.5 In vivo muscle function

Torque of the posterior hindlimb compartment was tested 8 weeks post‐VML as previously described (Greising et al., 2018; Southern et al., 2019). Mice were anesthetized (1.5%–2.0% inhaled isoflurane) and placed on their right side with the left hip and knee at 90° and left foot fixed to the footplate of the dual‐mode muscle lever system (300C‐LR; Aurora Scientific). Torque was measured after the peroneal nerve was severed to isolate the posterior compartment. Sciatic nerve stimulation was performed with platinum‐iridium percutaneous needle electrodes. Torque was measured over a range of frequencies (5, 10, 20, 30, 40, 60, 80, 100, 125, 150, 200 Hz). Data for twitch and maximal torque were normalized to body mass. Body temperature was maintained at 37°C during torque data collection.

### 2.6 Histologic analysis

Terminally, the EDL, soleus, and/or gastrocnemius muscles were harvested, frozen in 2‐methylbutane cooled by liquid nitrogen, and stored at −80°C until histological processing. Eight μm cross‐sections of the muscle were stained with Masson's trichrome according to procedures described previously (Dalske et al., 2021). Sections were imaged using a 20× objective (0.75 NA, 0.5 μm/pixel resolution) on a TissueScope LE brightfield slide scanner (Huron Digital Pathology). ImageJ was used to evaluate total myofiber number and average cross‐sectional myofiber area of each muscle sample (Schneider et al., 2012).

### 2.7 Biochemical analysis

The proximal portion of the gastrocnemius muscle was homogenized in 1:10 (mg/μL) 10 mM PO_4_ buffer with 1:100 protease phosphatase inhibitor using a Next Advance Bullet Blender with stainless steel beads. Protein content was measured using the Protein A280 setting on a NanoDrop One spectrophotometer (Thermo Fisher Scientific).

Twenty‐five to 50 μg of protein were loaded onto a 4%–20% Criterion TGX Stain‐Free Gel and blocked with 5% bovine serum albumin in 0.1% Tween TBS. The following primary antibodies were used at 1:1000 dilutions: Akt (Cell Signaling #9272), MyoD1 (Proteintech 18943‐1‐AP), Myf5 (Abcam ab125078), and Myostatin (Abcam ab124721). Corresponding host‐ and isotype‐specific secondary antibodies (Invitrogen; SA5‐10173, SA5‐10036, and SA5‐10176; 1:20,000 dilution) were used. Blots were first imaged (ChemiDoc System; Bio‐Rad) using the stain‐free setting for total lane protein evaluation and then imaged using the appropriate fluorescence channel to measure the protein band of interest. Image Lab software (Bio‐Rad) quantified total lane protein and band intensity. Bands were normalized to the total protein in each respective lane and compared across groups.

Evaluation of inflammatory, angiogenic, and fibrotic markers in the serum of the VML cohort mice was done via multiplex. Specifically, IL‐6, IL‐10, IL‐17A, and VEGFA (Millipore MCYTOMAG‐70K) and TGFβ1‐3 (Bio‐Rad #171W4001M) were evaluated according to manufacturer's technical specifications. Plates were read using a Bio‐Plex‐200 system (Bio‐Rad). Analytes were excluded from analysis if less than 65% of the total samples were below detection; IL‐17A and VEGFA did not meet this a priori threshold and were omitted from analysis.

### 2.8 Statistical analysis

Data were analyzed using GraphPad Prism (version 9.5.1, GraphPad Software) and JMP software (version 16.0 SAS Institute). Dependent variables were compared across treatment groups and considered significant at *p* ≤ 0.05. One‐way ANOVAs evaluated maximal torque and contractile parameters, muscle mass, myofiber number, ambulation, metabolic outcomes (e.g., MR, RER), and protein content. A two‐way ANOVA (group × time) evaluated body mass longitudinally. Where appropriate, Tukey's HSD post hoc determined specific differences between groups. Differences in distribution of myofiber cross‐sectional area were analyzed using a chi‐squared test and a Bonferroni post hoc (adjusted *p* ≤ 0.008). Data are presented as mean ± standard deviation (SD), with individual data points displayed per mouse.

## 3 RESULTS

### 3.1 Cohort 1: Injury naïve

**FIGURE 1:**
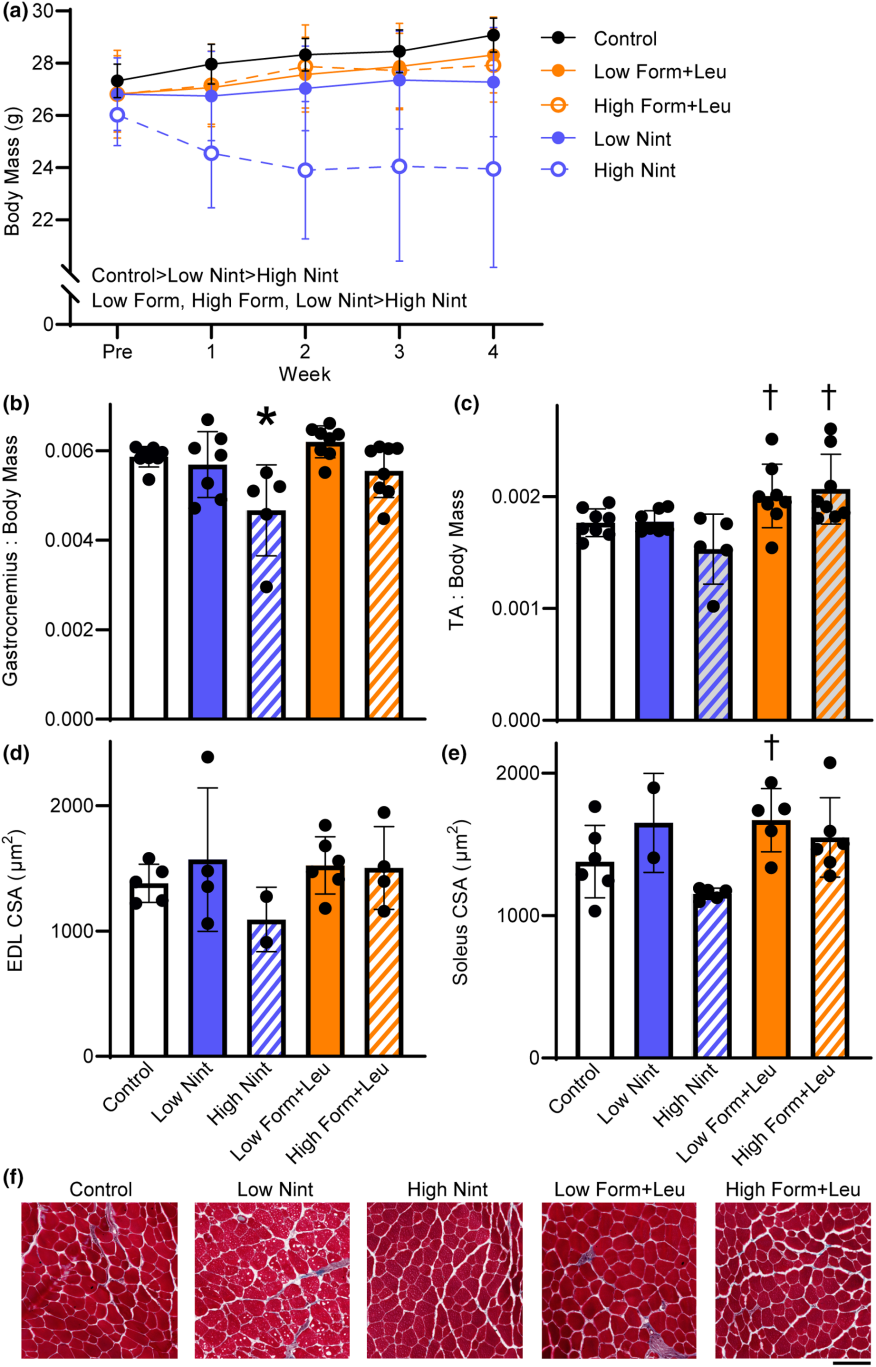
Treatment effects on body mass, muscle mass, and myofiber size were evaluated in the injury naïve cohort. (a) Over 4 weeks, body mass was significantly impaired with nintedanib, which was more pronounced with the high dose (main effect of group *p* < 0.001; main effect of time *p* = 0.545). (b) Gastrocnemius muscle mass (*p* = 0.002) and (c) tibialis anterior (TA) muscle mass (*p* = 0.003) normalized to body mass were less with high‐dose nintedanib. (d) Extensor digitorum longus (EDL) myofiber cross‐sectional (CSA) was not different between groups (*p* = 0.500), while (e) the high‐dose nintedanib impacted soleus muscle CSA (*p* = 0.018). (f) Representative EDL muscle sections, stained with Masson's Trichrome, across treatment groups and control. Scale bar is 100 μm. Data are mean ± SD; each data point represents an individual mouse. Significantly different from *control; ^†^high‐dose nintedanib.

The first cohort underwent 4 weeks of treatment to optimize dosing. Mice in both nintedanib groups had the lowest terminal body mass, which was more pronounced with the high dose (main effect of group *p* < 0.001; Figure 1a). Failure to gain weight longitudinally occurred in previous work investigating a porcine model of VML injury also (Corona et al., 2020). The high dose nintedanib significantly impacted gastrocnemius and TA muscle masses normalized to body mass (*p* ≤ 0.003; Figure 1b,c), but not EDL or soleus muscle masses normalized to body mass (*p* ≥ 0.170). The myofiber cross‐sectional area of the EDL muscle was not different across groups, but soleus myofiber cross‐sectional area was ~31% lower with the high dose of nintedanib compared to low dose formoterol plus leucine (*p* = 0.018; Figure 1d–f). As a result of the lower gastrocnemius and TA muscle masses (i.e., primary plantar‐ and dorsi‐ flexors muscles, respectively) and smaller myofiber size with the high dose of nintedanib treatment, the low dose of 6 mg/kg/day was selected for subsequent experiments. Both doses of formoterol plus leucine showed conservation of muscle mass similar to controls, or hypertrophy; therefore, the higher dose of 3 mg/kg/day plus 24 g/kg/day was selected for subsequent experiments.

### 3.2 Cohort 2: VML whole‐body metabolism

**FIGURE 2:**
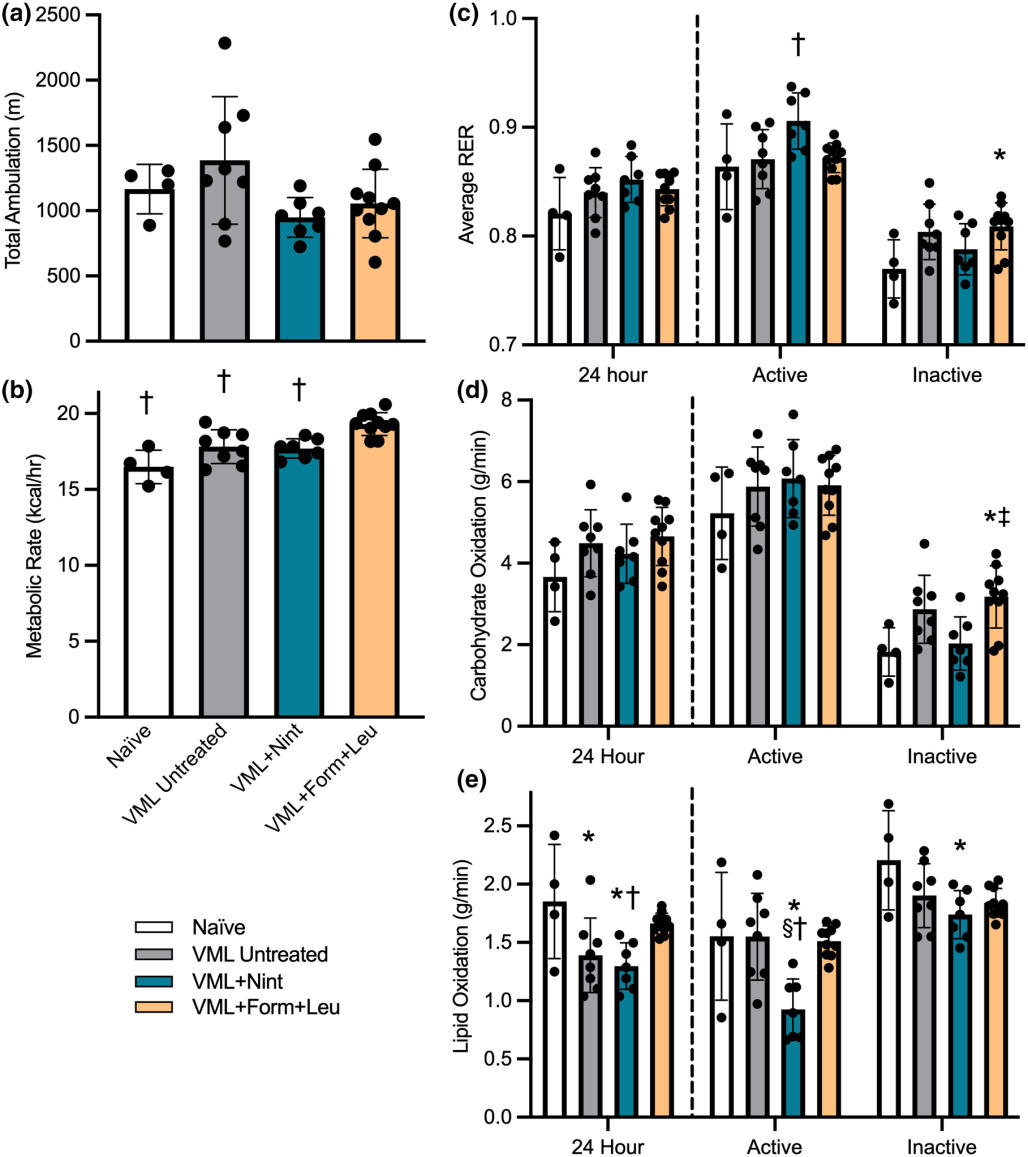
Physical activity and whole‐body metabolism were evaluated 6 weeks after VML. (a) Total ambulatory distance over 24 h was similar across groups (*p* = 0.070). (b) Formoterol plus leucine resulted in a higher 24‐h metabolic rate (*p* < 0.001). (c) Although 24‐h RER was similar across groups (*p* = 0.172), RER was elevated during the 12‐h active (*p* = 0.024) and inactive (*p* = 0.043) periods for nintedanib and formoterol plus leucine‐treated mice, respectively. (d) Carbohydrate oxidation was similar across groups over 24 h (*p* = 0.181) and during the 12‐h active period (*p* = 0.512) but was higher during the 12‐h inactive period following formoterol plus leucine treatment (*p* = 0.006). (e) VML‐untreated and nintedanib‐treated mice had a lower 24‐h lipid oxidation, while formoterol plus leucine treatment mitigated this decline (*p* = 0.005). Data are mean ± SD; each data point represents an individual mouse. Significantly different from *Naïve; ^§^VML untreated; ^‡^VML + nintedanib; ^†^VML + formoterol + leucine.

The effects of the optimized pharmaceutical doses of nintedanib or formoterol plus leucine were evaluated following VML injury and 8 weeks of treatments. Physical activity and whole‐body metabolism were evaluated at 6 weeks post‐injury. As expected, total ambulation was similar across groups (*p* = 0.070; Figure 2a). MR over 24 h was greatest in the formoterol plus leucine treatment group (16.5 kcal/h), ~17% and 8% higher than the injury naïve and VML untreated groups, respectively (*p* ≤ 0.001; Figure 2b), and over the 12‐h active and inactive (21.2 and 17.0 kcal/h, respectively) periods (*p* < 0.001). Although 24‐h RER and carbohydrate oxidation were not affected by treatment (*p* ≥ 0.172; Figure 2c,d), 24‐h lipid oxidation was greater with formoterol plus leucine treatment, similar to injury naïve controls, suggesting the intervention mitigates the VML‐induced impairment in lipid oxidation (*p* = 0.005; Figure 2e).

### 3.3 Cohort 2: VML muscle function and quality

**FIGURE 3:**
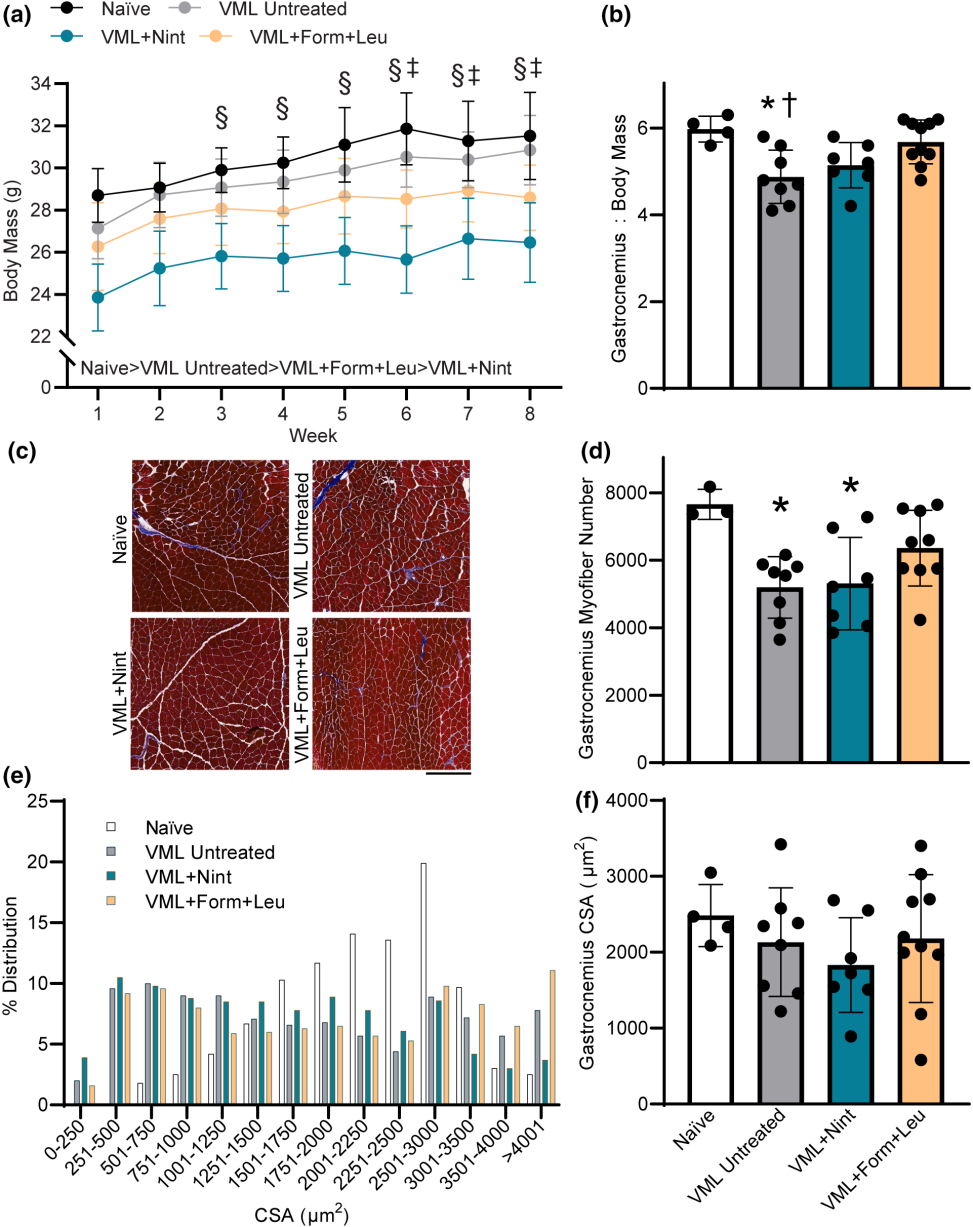
Treatment effects on body mass, muscle mass, and myofiber number and size were evaluated in the VML cohort. (a) Body mass increased over 8 weeks for all experimental groups but was smaller in both treatment groups (main effect of time *p* < 0.001; main effect of treatment *p* < 0.001). (b) Formoterol plus leucine‐treated mice had a higher gastrocnemius muscle mass normalized to body mass than VML‐untreated mice (*p* = 0.004). (c) Representative trichrome‐stained mid‐belly gastrocnemius muscle sections across groups; scale bar is 500 μm. (d) Mid‐belly gastrocnemius myofiber number following VML (*p* = 0.009). (e, f) Gastrocnemius myofiber average cross‐sectional area (CSA) distribution was different in all experimental groups compared to injury naïve, and in nintedanib‐treated versus formoterol (*p* < 0.001), but mean CSA was similar across groups (*p* = 0.534). Data are mean ± SD; each data point represents an individual mouse. Significantly different than ^**§**^week 1; ^‡^week 2; *naïve; ^†^VML + formoterol + leucine.

Across 8 weeks, mice in all groups gained body mass as expected. Injury naïve controls had the highest terminal body mass while VML‐injured, nintedanib‐treated mice had the lowest (*p* < 0.001; Figure 3a). Gastrocnemius mass normalized to body mass of VML untreated mice was less than that of controls, but both pharmacologic treatments attenuated this loss (*p* = 0.004; Figure 3b). Terminally, VML untreated and nintedanib‐treated mice had lower maximal isometric torque, but this deficit was attenuated with formoterol plus leucine treatment (*p* = 0.016; Table 1). There were no contractile differences across groups (*p* ≥ 0.093) except for the time to peak twitch and the average relaxation rate (−dP/dt) after achieving maximal torque (Table 1). All experimental groups, regardless of treatment, had a lower average relaxation rate, suggesting altered calcium homeostasis following VML which may reflect slower actin‐myosin cross‐bridge detachment that was not remedied by treatment (*p* = 0.005; Table 1).

**TABLE 1:** In vivo muscle contractile parameters.

	Injury naïve	VML untreated	VML + nintedanib	VML + formoterol + leucine	
	*n* = 4	*n* = 8	*n* = 7	*n* = 10	One‐way ANOVA *p*‐value
Gastrocnemius mass (mg)	188.0 ± 9.8	150.9 ± 19.4^a^	135.6 ± 16.3^a^ ^,^ ^c^	162.3 ± 21.7	0.001
Gastrocnemius protein content (mg/mL)	10.0 ± 1.1	9.8 ± 0.7	9.2 ± 1.3	10.2 ± 0.6	0.285
40 Hz/peak (%)	55.7 ± 4.4	52.3 ± 7.3	48.5 ± 6.0	57.4 ± 9.0	0.132
60 Hz/peak (%)	78.7 ± 5.0	78.8 ± 4.0	75.5 ± 8.1	82.0 ± 7.9	0.335
Time to peak twitch (s)	0.025 ± 0.001	0.024 ± 0.001	0.021 ± 0.002^a^ ^,^ ^b^ ^,^ ^c^	0.025 ± 0.001	0.001
Time to peak force (s)	0.089 ± 0.013	0.078 ± 0.009	0.074 ± 0.021	0.082 ± 0.014	0.453
½ Relaxation time (s)	0.046 ± 0.003	0.049 ± 0.006	0.045 ± 0.004	0.050 ± 0.003	0.280
+dP/dt (mN m/s)	331.5 ± 93.0	239.0 ± 28.6	236.6 ± 144.7	248.7 ± 44.7	0.093
−dP/dt (mN m/s)	−475.7 ± 100.6	−275.8 ± 58.8^a^	−236.6 ± 144.7^a^	−311.6 ± 83.6^a^	0.005
Maximal isometric torque (mN m/kg)	609.6 ± 109.6	356.3 ± 55.7^a^	350.2 ± 165.2^a^	406.7 ± 148.4	0.016

*Note*: Data are mean ± SD.

^a^ Significantly different than injury naïve.

^b^ VML untreated.

^c^ VML + Formoterol + Leucine.

Histologic evaluation of the VML‐injured gastrocnemius muscle supported the VML‐induced loss of myofibers at the muscle mid‐belly (*p* = 0.009; Figure 3c,d). While nintedanib treatment did not impact the total loss of myofibers after VML, formoterol plus leucine treatment resulted in greater myofiber number, or potentially supported myofiber regeneration. The distribution of gastrocnemius myofiber size was shifted leftward for all VML‐injured mice compared to naïve mice (*p* < 0.001). Although VML untreated mice had a similar distribution to nintedanib‐ and formoterol plus leucine‐ treated mice (*p* ≥ 0.030), nintedanib‐treated mice had a greater leftward shift compared to formoterol plus leucine treated mice (*p* < 0.0001; Figure 3e). However, there was no difference in the average gastrocnemius myofiber cross‐sectional area across groups (*p* = 0.534; Figure 3f).

**FIGURE 4:**
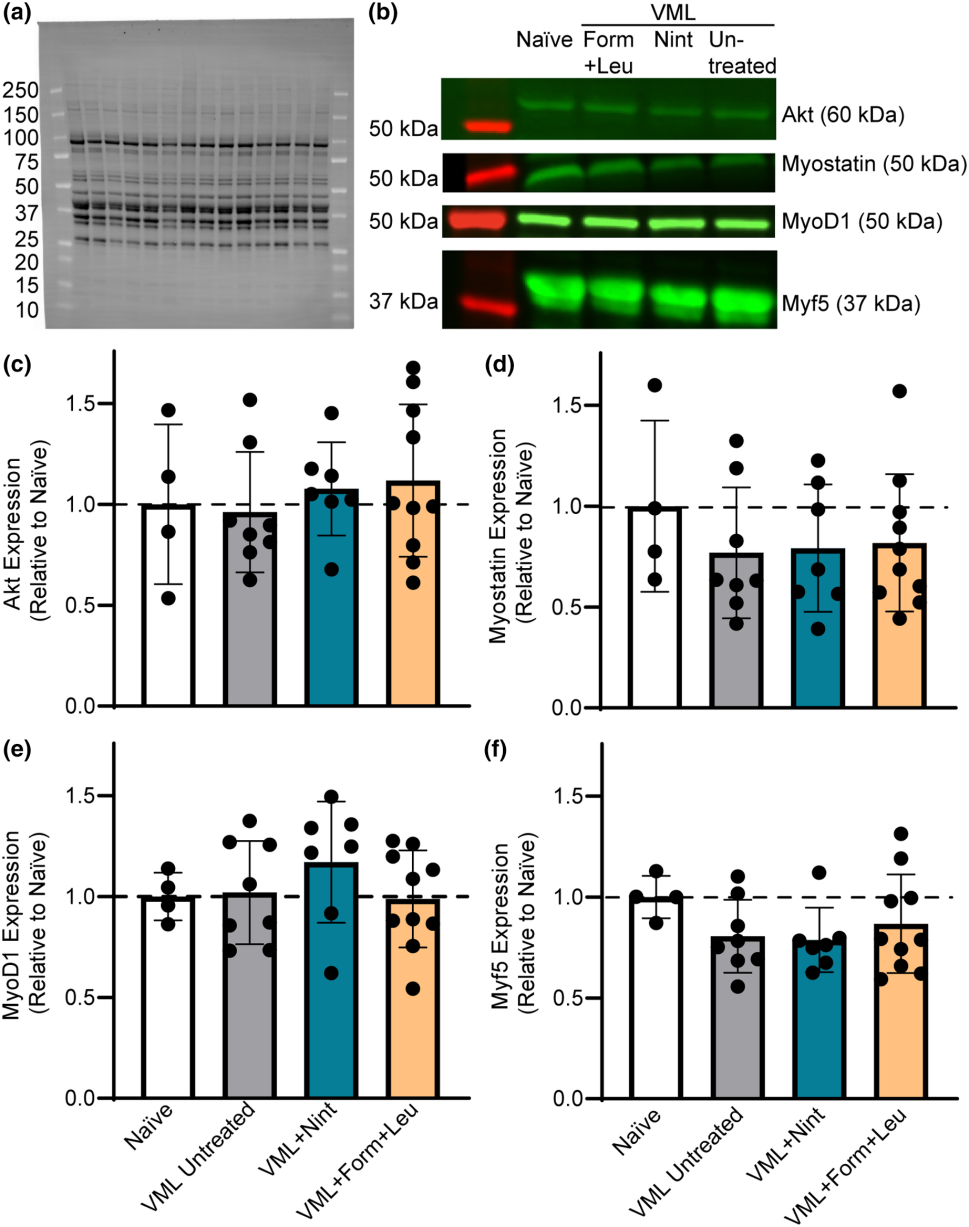
Evaluation of markers related to myogenesis and atrophy were measured 8 weeks following VML injury. (a) Representative stain‐free blot image displaying total lane protein with molecular weights of protein markers noted. (b) Corresponding fluorescent bands for each group and molecular weight for each marker. Bands were normalized to total protein in each respective lane to quantify relative protein expression compared to naïve. There were no differences in gastrocnemius muscle protein content of (c) Akt (*p* = 0.768), (d) myostatin (*p* = 0.723), (e) MyoD1 (*p* = 0.493), or (f) Myf5 (*p* = 0.383) across groups. Data are mean ± SD; each data point represents an individual mouse.

Circulating markers were evaluated to quantify systemic inflammatory responses affecting the overall cellular environment following VML. There were no statistical differences in circulating IL‐6 or IL‐10 across groups (Table 2); however, there was an inclination for upregulation, especially in the VML untreated group. Circulating TGFβ1‐3 levels were measured to specifically determine if nintedanib impacted broad changes in fibrotic signaling. Nintedanib's mechanism of action is downstream of TGFβ signaling, and while there was a slight trend to lower TGFβ1 with both nintedanib and formoterol, this was not significant (Table 2). The levels of TGFβ2 and TGFβ3 were similar across experimental groups (Table 2). Myogenic and atrophic signaling factors relevant to muscle protein synthesis and degradation were evaluated in the proximal gastrocnemius muscle. Independent of treatment, there were no differences in total protein content (*p* = 0.285; Table 1) or protein content of the myogenic markers Akt, MyoD and Myf5, or myostatin (*p* ≥ 0.383; Figure 4).

**TABLE 2:** Circulating factors.

	Injury naïve	VML untreated	VML + nintedanib	VML + formoterol + leucine	One‐way ANOVA *p*‐value
IL‐6 (pg/mL)	9.2 ± 1.6	38.0 ± 36.3	17.9 ± 6.6	16.4 ± 7.9	0.289
IL‐10 (pg/mL)	17.0 ± 8.6	46.7 ± 30.9	32.4 ± 17.0	17.1 ± 6.7	0.469
TGFβ1 (pg/mL)	3570.0 ± 2275.1	3638.8 ± 2197.1	2066.4 ± 1406.0	1946.0 ± 1101.1	0.162
TGFβ2 (pg/mL)	310.9 ± 179.9	316.1 ± 185.8	274.1 ± 153.5	215.2 ± 112.4	0.563
TGFβ3 (pg/mL)	46.0 ± 13.0	44.6 ± 14.7	35.3 ± 13.3	32.3 ± 8.7	0.137

*Note*: Data are mean ± SD.

## 4 DISCUSSION

VML injuries continue to be a significant clinical issue, due in part to lack of effective strategies to treat the loss of soft tissue and function, leading to lifelong impairment and disability. A possible strategy to target functional impairments is to repurpose currently FDA approved pharmaceuticals to promote regenerative and repair mechanisms in the muscle remaining after VML injury. This work evaluated two pharmaceuticals, including an anti‐fibrotic, nintedanib, and a muscle hypertrophy stimulant, formoterol combined with the essential amino acid leucine. Formoterol combined with leucine treatment after VML mitigated the VML‐induced loss of maximal isometric torque and gastrocnemius muscle mass and myofiber number, while enhancing MR. In contrast, nintedanib had no impact on maximal torque, muscle mass, and myofiber number, but did not further exacerbate the skeletal muscle phenotype after VML.

As a β_2_ adrenergic receptor agonist, formoterol stimulates skeletal muscle hypertrophy by stimulating intracellular signaling for myogenesis, including activation of the PI3K/Akt/mTOR pathway, while simultaneously mitigating atrophic signaling by inhibiting the FoxO family of transcription factors and therefore downstream atrophic factors (Joassard et al., 2013). Increased expression of related myogenic markers and decreased expression of atrophic markers are evident up to 3 weeks following formoterol treatment post‐spinal cord injury, corresponding with increased muscle mass and function (Ryall et al., 2006; Scholpa, Simmons, et al., 2019). Similarly, formoterol promoted muscle mass and myofiber regeneration up to 4 weeks following administration in *mdx* and wildtype mice (Harcourt et al., 2007; Ryall et al., 2006), as well as atrophy attenuation in rodents with cancer cachexia (Gomez‐SanMiguel et al., 2016; Kenley et al., 2008; Penna et al., 2019; Salazar‐Degracia et al., 2018). Recently in models of VML injury, (McFaline‐Figueroa et al., 2022; Raymond‐Pope et al., 2023) formoterol enhanced injured muscle mass, maximal torque production, and mitochondrial oxygen consumption, indicating improvements in functional muscle regeneration and oxidative capacity of the muscle remaining after injury. We hypothesized the addition of leucine to formoterol would enhance muscle mass and function following injury by stimulating the major regulator of protein synthesis, mTOR (Pereira et al., 2014; Zumbaugh et al., 2022). Herein, differences in myogenic marker protein expression were not observed across treatments; however, it is possible that myogenic signaling was transiently upregulated following injury with formoterol plus leucine treatment, which led to mass and contractile improvements in the injured gastrocnemius muscle chronically following VML. Indeed, treatment with formoterol plus leucine enhanced gastrocnemius mass as a result of a higher myofiber number, rather than a larger myofiber size, contributing to improved maximal force production that was similar to that of uninjured muscle.

Fibrotic formation after skeletal muscle injury replaces functional or parenchymal tissue with connective tissue (Wynn, 2008). This prevents full recovery of muscle function, limits cell migration, and disrupts biomechanical properties and structure of the muscle remaining (Gardner et al., 2020). Various anti‐fibrotic drugs, including nintedanib, have been evaluated in models of skeletal muscle injury. For example, losartan, an anti‐hypertensive and anti‐fibrotic pharmaceutical that inhibits angiotensin II type 1 receptor (AT1) and therefore blocks transforming growth factor beta 1 (TGF‐β1) and connective tissue growth factor (CCN2/CTGF), improved myofiber regeneration and function in injury models of contusion and laceration (Bedair et al., 2008; Kobayashi et al., 2013). However, these effects were not apparent following a more traumatic injury such as VML (Garg et al., 2014). As an intracellular tyrosine kinase inhibitor, nintedanib acts as an anti‐fibrotic and anti‐inflammatory pharmaceutical agent approved to treat idiopathic pulmonary fibrosis. Nintedanib inhibits fibrosis by targeting FGF, PDGF, and VEGF receptors at their intracellular ATP binding domains (Wollin et al., 2015). Nintedanib has been noted to delay and reverse the progression of fibrotic tissue accumulation in idiopathic pulmonary fibrosis (Wollin et al., 2015) and in the muscle of *mdx* mice (Pinol‐Jurado et al., 2018). Following VML injury, nintedanib prevented pathologic fibrotic tissue development and injury‐induced muscle stiffness; (Corona et al., 2020) however, it negatively impacted absolute muscle function and myofiber size, while force normalized to whole muscle size was not impacted. Similarly, herein, nintedanib was not able to improve force production after VML, likely due to a lower myofiber number and a leftward shift in myofiber size distribution. Mitigating fibrotic tissue deposition can prevent aberrant alterations in muscle structure and the microenvironment (Lieber & Ward, 2013). It is possible that a treatment regimen which incorporates nintedanib in the early phase following VML, with a myogenic promoter (e.g., β_2_ adrenergic receptor agonist) administered thereafter, could improve both the fibrotic and myogenic aspects of functional skeletal muscle regeneration chronically.

A recently overlooked aspect of VML injury is the maladaptive local muscle and whole‐body metabolic consequences of injury. Metabolic inflexibility, a blunted diurnal RER response characterized by a reduced ability to switch between carbohydrates and fats as fuel, ensues following VML (Dalske et al., 2021; Raymond‐Pope et al., 2023). This maladaptation is similar to observations following prolonged physical inactivity and in disease (e.g., Type II diabetes) (Rynders et al., 2018). Herein, although 24‐h RER was not altered, lipid oxidation was lower for both VML untreated and nintedanib‐treated mice, a characteristic also noted in individuals with obesity and metabolic disease (Rogge, 2009). However, formoterol plus leucine mitigated this decline, an observation supported by research indicating β_2_ adrenergic receptor agonists stimulate lipolysis (Hostrup & Onslev, 2022). Given that formoterol enhances mitochondrial biogenesis through its stimulation of PGC‐1α, (Pearen et al., 2009; Scholpa, Williams, et al., 2019) similar to effects of aerobic exercise, the resulting increased mitochondrial volume may shift substrate utilization from carbohydrates to lipids (Warren et al., 2020). This shift could result from an increased number of mitochondrial free fatty acid transporters (e.g., carnitine palmitoyltransferase; CPT‐I) and β‐oxidation enzymes for free fatty acid uptake and oxidation, respectively. In addition to enhanced lipid oxidation, formoterol plus leucine treated mice had a higher MR, consistent with previous observations for β_2_ adrenergic receptor agonists (Hostrup & Onslev, 2022), likely influenced by a higher myofiber number and muscle mass. Indeed, a higher amount of lean muscle mass (i.e., fat‐free mass) is the primary contributing factor to increased MR, explaining up to 83% of its variability (Buchholz et al., 2001; Sparti et al., 1997; Speakman & Selman, 2003). Observations suggest treatment with formoterol combined with leucine beneficially mitigates both the local muscle and whole‐body metabolic responses that result from VML injury (McFaline‐Figueroa et al., 2022; Raymond‐Pope et al., 2023).

## 5 CONCLUSION

To continue evaluating potential therapeutic options for patients after VML, future investigations should focus on promoting myogenesis and metabolism using β_2_ adrenergic receptor agonists such as formoterol. The beneficial improvements in functional skeletal muscle regeneration (i.e., myofiber number and force) and metabolism with formoterol plus leucine treatment support its use in future translational investigations of VML. Further research is warranted to explore adjunctive β_2_ adrenergic agonists loaded into a scaffold for local muscle administration of the drug, with or without rehabilitative exercise (e.g., running). In addition to the optimal dose of formoterol plus leucine identified herein, it is important to identify the optimal timing of initiation and duration of a combined regenerative rehabilitative approach to enhance functional muscle regeneration and metabolic adaptations.

## AUTHOR CONTRIBUTIONS

Experiments were conducted in the laboratory of Sarah M. Greising. Jarrod A. Call and Sarah M. Greising were involved in conception or design of the work. Shefali R. Bijwadia, Christiana J. Raymond‐Pope, Alec M. Basten, Thomas J. Lillquist, Mason T. Lentz, Jarrod A. Call, and Sarah M. Greising were involved in acquisition, analysis, or interpretation of data for the work. Shefali R. Bijwadia, Christiana J. Raymond‐Pope, Jarrod A. Call, and Sarah M. Greising were involved in drafting of the work or revising it critically for important intellectual content. All authors approved the final version of the manuscript. All authors agree to be accountable for all aspects of the work in ensuring that questions related to the accuracy or integrity of any part of the work are appropriately investigated and resolved. All persons designated as authors qualify for authorship, and all those who qualify for authorship are listed.

## FUNDING INFORMATION

Funding through the Congressionally Directed Medical Research Program, Clinical & Rehabilitative Medicine Research Program: W81XWH‐20‐10,885 (JAC and SMG). SRB was supported in part by a University of Minnesota Foundation Medical Student Research Grant. The funders had no role in study design, data collection and analysis, decision to publish, or preparation of the manuscript. Opinions, interpretations, conclusions and recommendations are those of the authors and are not necessarily endorsed by the Department of Defense.

## CONFLICT OF INTEREST STATEMENT

The authors declare that they have no conflict of interests.

## Data Availability

The datasets used and/or analyzed during the current study are primarily presented in the current manuscript and are available from the corresponding author on request.
